# Immediate histopathological outcomes according to Aptima HPV E6/E7 mRNA genotype, age, and menopausal status in women with ASC-H/HSIL cytology

**DOI:** 10.3389/fonc.2026.1952454

**Published:** 2026-10-26

**Authors:** Pan Liu, Zhina Liu, Yuxing Yang, Bing Du, Hongling Zuo

**Affiliations:** 1 Department of Obstetrics and Gynecology, Hebei Reproductive Health Hospital, Shijiazhuang, Hebei, China; 2 Department of Obstetrics and Gynecology, Peking University Third Hospital Chongli, Zhangjiakou, Hebei, China; 3 Department of Obstetrics and Gynecology, The Second Hospital of Hebei Medical University, Shijiazhuang, Hebei, China

**Keywords:** Aptima HPV, ASC-H/HSIL, CIN2+, E6/E7 mRNA, histopathological outcomes

## Abstract

**Objective:**

To examine the associations of Aptima HPV E6/E7 mRNA genotype, age, and menopausal status with immediate histopathological outcomes in women with ASC-H/HSIL cytology.

**Methods:**

We retrospectively analyzed 330 women aged 20–84 years with ASC-H/HSIL cytology who underwent Aptima HPV testing, colposcopy-guided biopsy, and histopathological examination. Histopathological outcomes were compared according to hrHPV genotype, age, and menopausal status. Multivariable logistic regression was used to evaluate the associations of hrHPV genotype, age, and menopausal status with CIN2 +.

**Results:**

Among women with ASC-H/HSIL cytology, 38.2% had cervical precancerous lesions and 24.5% had malignant lesions. HPV16 and the other 11 hrHPV types were associated with a higher prevalence of CIN2/3, while HPV16-positive women had the highest prevalence of SCC. Both AIS cases occurred in the HPV18/45-positive group, although this subgroup was small. CIN2/3 prevalence decreased with age, whereas SCC prevalence increased. In the multivariable analysis, HPV16, HPV18/45, and the other 11 hrHPV types were independently associated with CIN2+, whereas age and menopausal status were not.

**Conclusions:**

Histopathological outcomes among women with ASC-H/HSIL cytology differed according to hrHPV genotype. HPV16 positivity was strongly associated with cervical precancerous and malignant lesions. Age and menopausal status showed differences in unadjusted analyses but were not independently associated with CIN2 +.

## 1 Introduction

Cervical cancer is a highly prevalent gynecological malignancy in clinical practice, posing a severe threat to women’s lives and health worldwide. In 2022, there were an estimated 661, 000 new cases and approximately 348, 000 deaths from cervical cancer globally, ranking it fourth among female malignancies in both incidence and mortality (1). The situation in China is even more severe, with its incidence and mortality rates consistently topping the list of gynecological malignancies for an extended period (2). Geographically, the disease burden is highly uneven: the incidence (19.3 per 100, 000) and mortality (12.4 per 100, 000) in low- and medium-Human Development Index (HDI) countries are significantly higher than those in very high-HDI countries (incidence: 12.1 per 100, 000; mortality: 4.8 per 100, 000). In China, a positive shift has emerged in the overall incidence trend of cervical cancer. Since 2008, the rate of increase in both incidence and mortality has slowed down significantly, with a particularly notable declining trend observed among young women. This confirms that China’s comprehensive prevention and control strategies have achieved phased results (2). Nevertheless, cervical cancer remains the primary gynecological malignancy threatening the health of Chinese women.

Cervical cancer is a malignant tumor characterized by progressive development, predominantly arising from the long-term progression of precancerous lesions. This pathological feature provides a critical window of opportunity for early screening and clinical intervention. Early detection of precancerous lesions and accurate risk assessment constitute the core strategies for reducing the incidence and mortality of cervical cancer, while holding significant clinical and public health value. Currently, cervical cytology serves as the primary initial screening modality in China’s national “two cancers” screening program for women at the grassroots level. Cytology has limited sensitivity and interobserver variability, therefore combined hrHPV testing is recommended by ASCCP. Test results heavily depend on the subjective experience of pathologists, often leading to ambiguous diagnoses or inconclusive findings that necessitate repeat testing or further examinations. Consequently, its relatively low screening sensitivity and poor inter-observer diagnostic concordance severely compromise the quality and efficiency of grassroots screening programs (3). In recent years, with advances in molecular epidemiological research, persistent hrHPV infection has been firmly established as the central etiological factor in cervical carcinogenesis (4, 5). Based on this pivotal finding, clinical cervical cancer screening strategies are shifting from sole cytomorphological assessment to combined molecular biological testing. In 2019, the American Society for Colposcopy and Cervical Pathology (ASCCP) issued official guidelines formally recommending cervical cytology combined with hrHPV testing as the standardized screening protocol to address the technical shortcomings of cytology-alone screening (6). Notably, substantial variations exist in the carcinogenic risks associated with different hrHPV genotypes (7), and currently available detection technologies each have distinct strengths and weaknesses in terms of sensitivity and specificity, necessitating judicious selection based on population characteristics. Since 2001, the U.S. Food and Drug Administration (FDA) has approved five HPV assays, comprising four DNA-based tests and one mRNA-based test (7). Unlike DNA-based assays, the Aptima assay detects transcriptionally active E6/E7 mRNA and therefore provides higher specificity for clinically significant lesions (8, 9). The Aptima HPV (AHPV) assay employs mRNA detection to specifically identify transcriptionally active viruses, thereby offering a higher positive predictive value and is considered more appropriate as a primary method for cervical cancer screening (10).

Although the majority of abnormal cervical cytology results do not progress to cancer, individuals with a cytological diagnosis of atypical squamous cells, cannot exclude high-grade squamous intraepithelial lesion (ASC-H) or high-grade squamous intraepithelial lesion (HSIL) face a substantially elevated risk of developing cervical intraepithelial neoplasia grade 2 or worse (CIN2+), with reported incidences reaching up to 53.76% and 82.07%, respectively (11). These patients carry a high risk of precancerous lesions and cancer and typically require immediate intervention (6, 12). Existing evidence suggests that risk stratification of patients with negative for intraepithelial lesion or malignancy (NILM) or atypical squamous cells of undetermined significance (ASC-US) cytology using AHPV testing helps optimize patient management strategies (13, 14). However, studies investigating the association between ASC-H/HSIL cytology and hrHPV infection—particularly those based on AHPV testing in Chinese populations—remain relatively scarce.

China continues to bear a heavy burden of cervical cancer. The underlying cause is the high prevalence of hrHPV infection and CIN2+ lesions among elderly women, which constitutes the primary driver of this public health challenge (15). Postmenopausal women experience hormonal fluctuations and immune senescence, compounded by the reactivation of latent HPV infections, all of which contribute to a markedly elevated risk of disease onset (16). Although clinical practice often identifies women under 50 as the high-incidence group, the proportion of confirmed cases in women over 65 years of age should not be overlooked. Moreover, with increasing life expectancy, this proportion is expected to continue rising (17). Age-related retraction of the cervical transformation zone into the endocervical canal makes cervical sampling and lesion detection more challenging, particularly in older women (18). Concurrently, the coverage and frequency of cervical cancer screening remain suboptimal among elderly women, leading to a systematic underestimation of the true disease burden, and their long-term prognosis is significantly poorer than that of younger patients (16). Although the American Cancer Society designates 65 years as the upper age limit for routine cervical cancer screening, multiple clinical studies have documented a substantial number of cervical cancer cases diagnosed after the age of 69, indicating that the current screening age cutoff fails to fully capture the risk in elderly women (17). Against the backdrop of China’s accelerating population aging and the steadily expanding postmenopausal female population, accurately assessing the potential risk of cervical cancer and high-grade precancerous lesions in this specific demographic, and formulating tailored prevention, control, and clinical management strategies, have become urgent priorities in the field of gynecologic oncology.

Therefore, this retrospective study aimed to examine the associations of hrHPV genotype, age, and menopausal status with immediate histopathological outcomes in women with ASC-H/HSIL cytology.

## 2 Materials and methods

### 2.1 Study population

From January 2021 to February 2024, a total of 511 female cases diagnosed with ASC-H/HSIL were recorded in the ThinPrep Cytology Test (TCT) database at the Second Hospital of Hebei Medical University. Of the 511 women initially identified, 181 were excluded: 37 had no Aptima HPV testing, 111 had no colposcopy-guided cervical biopsy or definitive histopathological diagnosis, 7 had a history of hysterectomy or cervical malignancy, 24 had another malignancy or prior pelvic radiotherapy/chemotherapy, and 2 had an autoimmune disease. Exclusion reasons were assigned hierarchically and mutually exclusively. The remaining 330 women were included in the final analysis (**Figure 1**). As illustrated in **Figure 1**, 330 women aged 20 to 84 years with abnormal cytological results of ASC-H/HSIL and concurrent AHPV test results underwent follow-up biopsy in accordance with the ASCCP guidelines.

**Figure 1:**
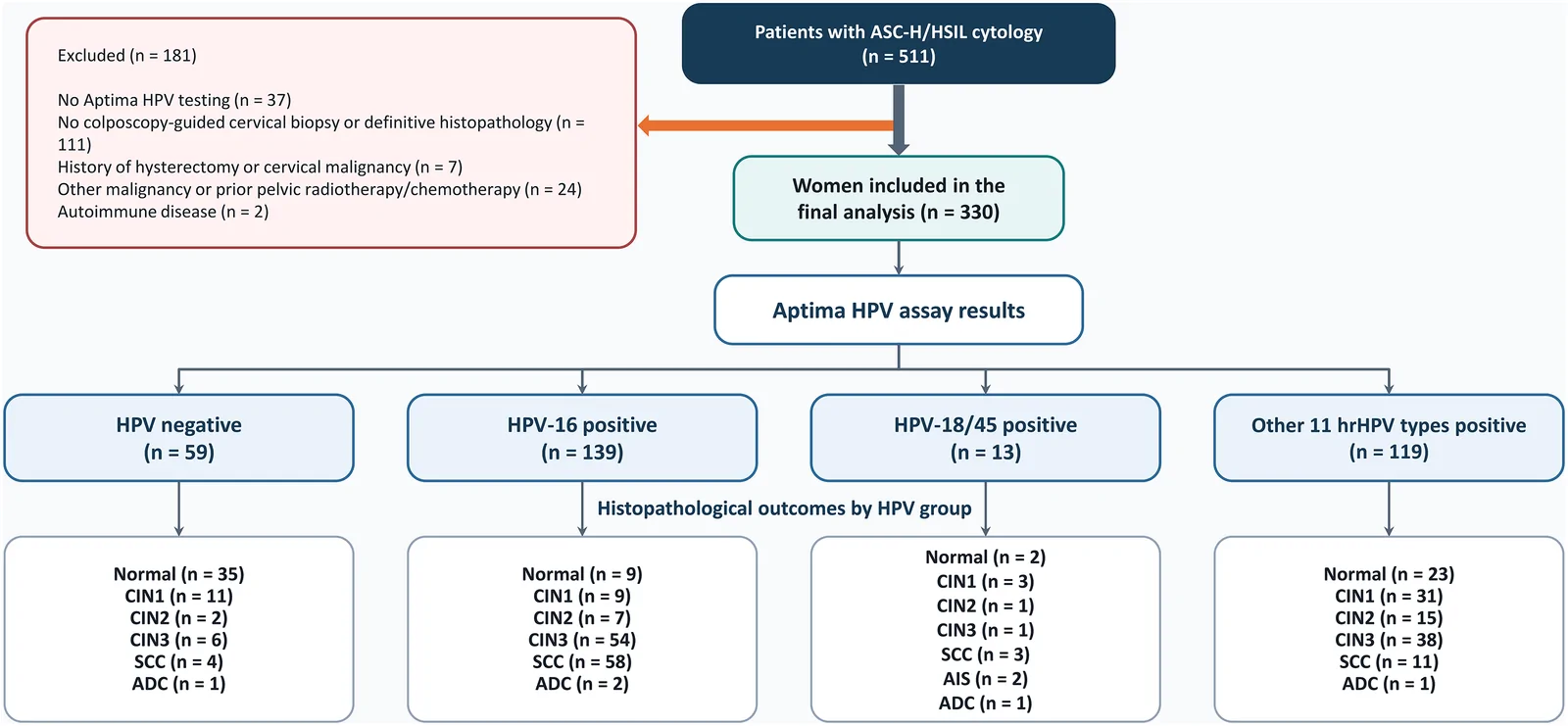
Flow chart of selection criteria of participants.

Menopausal status was determined from the menstrual history documented in the medical records. Natural menopause was defined retrospectively as at least 12 consecutive months of amenorrhea without another obvious physiological or pathological cause and in the absence of clinical intervention. For the binary analysis, women who did not meet this criterion were classified as premenopausal (19).

### 2.2 Inclusion and exclusion criteria

Inclusion criteria: (1) Women with a history of sexual activity who were not pregnant; (2) Those who underwent both AHPV testing and TCT, with cytological results of ASC-H/HSIL; (3) Those with a definitive pathological diagnosis from cervical biopsy.

Exclusion criteria: (1) Individuals with incomplete clinical data or poor follow-up compliance; (2) Those with a history of hysterectomy or malignant tumors; (3) Those with a history of other malignancies or prior pelvic radiotherapy/chemotherapy; (4) Those with autoimmune diseases; (5) Those currently receiving hormone replacement therapy.

This study was approved by the Ethics Committee of the Second Hospital of Hebei Medical University.

### 2.3 TCT testing

TCT testing was performed using a fully automated ThinPrep 2000 liquid-based cytology processor, strictly following the standardized operating procedures outlined in the manufacturer’s instructions. Cytological results were interpreted and classified according to the modified 2001 version of The Bethesda System (TBS) reporting system (20). The specific diagnostic categories included: (1) Negative for intraepithelial lesion or malignancy (NILM); (2) Atypical squamous cells (ASC-US and ASC-H) and atypical glandular cells (AGC); (3) Low-grade squamous intraepithelial lesion (LSIL); (4) High-grade squamous intraepithelial lesion (HSIL); and (5) Squamous cell carcinoma (SCC) and adenocarcinoma (ADC). For the purpose of this study, a TCT result of ASC-H/HSIL was defined as a positive cytological finding.

### 2.4 HR-HPV E6/E7 mRNA testing

The nucleic acid amplification detection system comprised the APTIMA HPV HR assay and the APTIMA HPV 16 18/45 Genotype assay. The former was used for the qualitative detection of E6/E7 mRNA from 14 hrHPV types (16, 18, 31, 33, 35, 39, 45, 51, 52, 56, 58, 59, 66, and 68) without distinguishing individual genotypes. Specimens positive for hrHPV were subsequently tested using the genotype assay and categorized as HPV16-positive, HPV18/45-positive, or positive for other hrHPV types. An HPV18/45-positive result indicated the detection of HPV18 and/or HPV45; the assay could not distinguish between these two genotypes or establish their co-infection. Specimens positive for hrHPV but negative for HPV16 and HPV18/45 were classified as positive for the other 11 hrHPV types.

### 2.5 Histopathology

Cervical histopathology served as the reference standard. All tissue specimens were fixed in 10% neutral-buffered formalin, routinely processed, embedded in paraffin, sectioned at 4 μm, and stained with hematoxylin and eosin. The specimens were evaluated by two pathologists, each with at least 5 years of experience in gynecological pathology, according to the World Health Organization Classification of Female Genital Tumors (2020) (21).

Histopathological diagnoses were classified as follows: (1) normal/inflammation; (2) low-grade squamous intraepithelial lesion (LSIL), encompassing cervical intraepithelial neoplasia grade 1 (CIN1); (3) high-grade squamous intraepithelial lesion (HSIL), encompassing CIN2 and CIN3; (4) squamous cell carcinoma (SCC); (5) adenocarcinoma *in situ* (AIS); and (6) adenocarcinoma (ADC). In cases suspected of CIN2, immunohistochemical staining for p16 and Ki-67 was routinely performed to reduce diagnostic variability between CIN1 and CIN2. Diffuse block-positive p16 staining together with Ki-67-positive cells extending above the lower one-third of the epithelium supported a diagnosis of HSIL (CIN2).

A hierarchical composite reference standard was used to determine the final histopathological diagnosis. For women who underwent both colposcopy-directed biopsy and subsequent excisional or surgical treatment during the same episode of care, including loop electrosurgical excision procedure, cold-knife conization, or hysterectomy, the highest histopathological grade identified across all available specimens was used as the final diagnosis. For women who underwent biopsy alone, the biopsy result was used. Overall, the final diagnoses of 155 women (47.0%) incorporated findings from excisional or surgical specimens, and 14 of these women had a higher histopathological grade in the excisional or surgical specimen than in the preceding biopsy. When biopsies were obtained from different sites or on multiple occasions, the highest recorded histopathological grade was likewise used as the final diagnosis.

For consistency across the analyses, CIN2+ was defined as CIN2, CIN3, AIS, SCC, or ADC. Squamous HSIL+ was defined as CIN2, CIN3, or SCC, whereas AIS+ was defined as AIS or ADC. Precancerous lesions comprised CIN2, CIN3, and AIS, while malignant lesions comprised SCC and ADC.

### 2.6 Statistical methods

Continuous variables were summarized as means with standard deviations and compared using the independent-samples t test. Categorical variables were summarized as numbers and percentages and compared using Pearson’s chi-square test or Fisher’s exact test, as appropriate. Age was additionally dichotomized at 30, 40, 50, and 60 years in exploratory sensitivity analyses to examine whether the observed age-related patterns were consistent across alternative thresholds. These overlapping cut-off analyses were not treated as independent confirmatory hypothesis tests; the reported P values were unadjusted for multiple comparisons and should therefore be interpreted descriptively. Age, menopausal status, and hrHPV genotype were prespecified as candidate covariates based on the study objectives and their clinical relevance. All three variables were entered simultaneously into the multivariable binary logistic regression models using the enter method, without univariable P-value screening or stepwise variable selection. Age was modeled as a continuous variable, menopausal status as a binary variable, and hrHPV genotype as a categorical variable, with hrHPV-negative women as the reference group. Potential multicollinearity among the candidate variables was assessed using variance inflation factors (VIFs). Although age and menopausal status were correlated, their VIFs were 2.35 and 2.37, respectively, indicating no problematic multicollinearity. Both variables were therefore retained because they had been prespecified on the basis of clinical relevance. The primary outcome was CIN2+, defined as CIN2, CIN3, AIS, SCC, or ADC; normal/inflammatory findings and CIN1 were classified as the negative outcome. Secondary endpoints included squamous HSIL+ (CIN2, CIN3, or SCC), AIS+ (AIS or ADC), CIN2/3, and SCC. Adjusted odds ratios and 95% confidence intervals were reported. All statistical tests were two-sided, and P < 0.05 was considered statistically significant.

## 3 Results

### 3.1 Demographic characteristics of women with ASC-H/HSIL cytology results

A total of 330 women with ASC-H or HSIL cytology who underwent cervical biopsy were included in this study. Of these, 169 (51.2%) had ASC-H cytology and 161 (48.8%) had HSIL cytology. As shown in **Table 1**, the age of the enrolled women ranged from 20 to 84 years, with a mean age of 44.75 ± 12.44 years. The mean age was 43.47 years in the ASC-H group and 46.11 years in the HSIL group, with no significant difference between the two groups (P = 0.055). The overall hrHPV positivity rate was 82.1%. The hrHPV positivity rate was significantly higher in women with HSIL than in those with ASC-H (94.4% vs. 70.4%, P < 0.001). Histopathological examination showed that 38.2% of women had precancerous lesions and 24.5% had malignant lesions. Furthermore, the distribution of histopathological outcomes differed significantly between the ASC-H and HSIL groups (P < 0.001), with women in the HSIL group showing a substantially higher proportion of malignant lesions than those in the ASC-H group.

**Table 1:** Demographic characteristics of patients with ASC-H and HSIL cytology.

Indicators	ASC-H (n=169)	HSIL (n=161)	Total(n=330)	*t/x (^2^)*	*P*
Age (year)	43.47 ± 11.64	46.11 ± 13.13	44.75 ± 12.44	-1.93	0.055
hrHPV infection					
Positive	119 (70.4%)	152 (94.4%)	271 (82.1%)	32.34	<0.001
Negative	50 (29.6%)	9 (5.6%)	59 (17.9%)		
Histopathological outcome					
Normal/inflammation	59 (34.9%)^*^	10 (6.2%)	69 (20.9%)	77.36	<0.001
LSIL (CIN1)	41 (24.3%)	13 (8.1%)	54 (16.4%)		
Precancerous lesions	50 (29.6%)	76 (47.2%)	126 (38.2%)		
Malignant lesions	19 (11.2%)^*^	62 (38.5%)	81 (24.5%)		

1. Normal/inflammation included histologically normal findings and inflammatory changes; LSIL included CIN1; precancerous lesions included CIN2, CIN3, and AIS; malignant lesions included SCC and ADC. 2. *P < 0.05 compared with the HSIL group.

### 3.2 Pathological distribution of hrHPV genotypes in women with ASC-H/HSIL

As shown in **Table 2**, among the 330 women with ASC-H/HSIL cytology, 59 (17.9%) were hrHPV-negative, 139 (42.1%) were positive for HPV16, 13 (3.9%) were positive for HPV18/45, and 119 (36.1%) were positive for the other 11 hrHPV types. Histopathological examination demonstrated distinct lesion distributions across different hrHPV genotypes. In the hrHPV-negative group, benign lesions predominated, with normal histology accounting for 59.3% of cases. In contrast, HPV16-positive women exhibited the highest proportions of CIN3 (38.8%) and SCC (41.7%), indicating a strong association with high-grade cervical lesions and invasive carcinoma. Among HPV18/45-positive women, histopathological findings were distributed across benign, precancerous, and malignant lesions, including the only two cases of AIS identified in this cohort. In women infected with the other 11 hrHPV types, CIN1 (26.1%) and CIN3 (31.9%) were the most frequently observed histopathological diagnoses.

**Table 2:** Distribution of HPV infection patterns among the women with ASC-H/HSIL cytology.

hrHPV genotype	Normal	CIN 1	CIN 2	CIN 3	SCC	AIS	ADC	Total
HPV negative	35 (59.3%)	11 (18.6%)	2 (3.4%)	6 (10.2%)	4 (6.8%)	0	1 (1.7%)	59 (17.9%)
HPV16	9 (6.5%)	9 (6.5%)	7 (5.0%)	54 (38.8%)	58 (41.7%)	0	2 (1.4%)	139 (42.1%)
HPV18/45	2 (15.4%)	3 (23.1%)	1 (7.7%)	1 (7.7%)	3 (23.1%)	2 (15.4%)	1 (7.7%)	13 (3.9%)
Other 11 types	23 (19.3%)	31 (26.1%)	15 (12.6%)	38 (31.9%)	11 (9.2%)	0	1 (0.8%)	119 (36.1%)
Total	69 (20.9%)	54 (16.4%)	25 (7.6%)	99 (30.0%)	76 (23.0%)	2 (0.6%)	5 (1.5%)	330

As shown in **Table 3**, the prevalence of CIN2/3 differed significantly among hrHPV genotype groups (P < 0.001). Women with HPV16 infection (43.9%) and those infected with the other 11 hrHPV types (44.5%) had significantly higher CIN2/3 prevalence than hrHPV-negative women (13.6%). Likewise, SCC was significantly more common in the HPV16-positive group (41.7%) than in both the hrHPV-negative (6.8%) and the other 11 hrHPV-positive (9.2%) groups (P < 0.001). For glandular lesions, AIS was observed exclusively in the HPV18/45-positive group (15.4%), resulting in a significantly higher prevalence than that in the other genotype groups (overall P = 0.001), whereas the prevalence of ADC did not differ significantly among the groups (P = 0.293).

**Table 3:** Distribution of HPV infection patterns among the women with ASC-H/HSIL cytology.

hrHPV genotype	Total	Squamous lesions						Glandular lesions			
		CIN2/3	*χ (^2^)*	*P*	SCC	*χ (^2^)*	*P*	AIS	*P*	ADC	*P*
HPV negative	59	8 (13.6%)	22.06	<0.001	4(6.8%)^b^	48.96	<0.001	0^c^	0.001	1(1.7%)	0.293
HPV16	139	61(43.9%)^a^			58(41.7%)			0^c^		2(1.4%)	
HPV18/45	13	2 (15.4%)			3 (23.1%)			2(15.4%)		1(7.7%)	
Other 11 types	119	53(44.5%)^a^			11(9.2%)^b^			0^c^		1(0.8%)	
Total	330	124(37.6%)			76(23.0%)			2 (0.6%)^c^		5(1.5%)	

^a^vs. hrHPV-negative group, *P* < 0.05; ^b^vs. hrHPV16 group, *P* < 0.05; ^c^vs. hrHPV18/45 group, *P* < 0.05.

### 3.3 Distribution of hrHPV genotypes across different age groups in women with ASC-H/HSIL

The distribution of hrHPV genotypes was further analyzed according to age group and menopausal status (**Table 4**). Among the 330 women with ASC-H/HSIL cytology, HPV16 was the most common genotype across all age groups, followed by the other 11 hrHPV types. Although the proportion of HPV16-positive cases appeared to increase with advancing age, particularly among women aged ≥55 years, no significant differences in hrHPV genotype distribution were observed across the different age groups (P = 0.183). In contrast, analysis according to menopausal status demonstrated significant differences in hrHPV genotype distribution (P = 0.010). Specifically, the hrHPV-negative rate was significantly higher in premenopausal women than in postmenopausal women (21.6% vs. 8.5%), whereas HPV16 infection was significantly more frequent in postmenopausal women than in premenopausal women (54.3% vs. 37.3%). No significant differences were observed in the prevalence of HPV18/45 or the other 11 hrHPV types between the two groups.

**Table 4:** Distribution of hrHPV genotypes across different age groups in women with ASC-H/HSIL.

Indicators	Total	hrHPV genotype				*χ (^2^)*	*P*
		HPV negative	HPV16	HPV18/45	Other 11 types		
Age							
<25	2	0	1 (50.0%)	0	1 (50.0%)		0.183
25-34	81	16 (19.8%)	29 (35.8%)	4 (4.9%)	32 (39.5%)		
35-44	91	23 (25.3%)	35 (38.5%)	3 (3.3%)	30 (33.0%)		
45-54	79	15 (19.0%)	31 (39.2%)	4 (5.1%)	29 (36.7%)		
55-64	53	3 (5.7%)	27 (50.9%)	2 (3.8%)	21 (39.6%)		
≥65	24	2 (8.3%)	16 (66.7%)	0	6 (25.0%)		
Menopausal status							
Premenopausal	236	51 (21.6%)^*^	88 (37.3%)^*^	9 (3.8%)	88 (37.3%)	11.43	0.010
Postmenopausal	94	8 (8.5%)	51 (54.3%)	4 (4.3%)	31 (33.0%)		
Total	330	59	139	13	119		

*vs. postmenopausal group, *P* < 0.05.

The distribution of histopathological outcomes according to age and menopausal status is presented in **Table 5** and **6**. Only two women were aged <25 years; both had CIN2/3, and neither had SCC, AIS, nor ADC. Because of the very small sample size, these patients were described separately and excluded from the age-stratified inferential analysis. Among the remaining 328 women, significant age-related differences were observed in the prevalence of CIN2/3 and SCC. The prevalence of CIN2/3 decreased progressively with increasing age and was significantly higher among women aged 25–44 years than among those aged ≥55 years, whereas SCC was significantly more frequent among women aged ≥55 years than among those aged 25–54 years (both P < 0.001). No significant age-related differences were observed for AIS (P = 0.224) or ADC (P > 0.999). Similarly, premenopausal women had a significantly higher prevalence of CIN2/3, whereas postmenopausal women had a significantly higher prevalence of SCC (both P < 0.001). Menopausal status was not significantly associated with AIS (P = 0.489) or ADC (P = 0.626). Exploratory sensitivity analyses using age cut-offs of 30, 40, 50, and 60 years showed generally consistent patterns, with a higher prevalence of CIN2/3 in younger women and a higher prevalence of SCC in older women, whereas no consistent differences were observed for glandular lesions (**Table 6**).

**Table 5:** Age-stratified histopathological outcomes in women with ASC-H/HSIL cytology.

Indicators	Total	Squamous lesions						Glandular lesions				
		CIN2/3	*χ (^2^)*	*P*	SCC	*χ (^2^)*	*P*	AIS	*P*	ADC	*P*	
Age			25.11	<0.001		65.04	<0.001		0.224		>0.999	
25~34	81	41 (50.6%)			7 (8.6%)			0 (0.0%)		1 (1.2%)		
35~44	91	43 (47.3%)			9 (9.9%)			0 (0.0%)		2 (2.2%)		
45~54	79	25 (31.6%)			17 (21.5%)			2 (2.5%)		1 (1.3%)		
55~64	53	10 (18.9%)^b^			29 (54.7%)^c^			0 (0.0%)		1 (1.9%)		
≥65	24	3 (12.5%)^ab^			14 (58.3%)^c^			0 (0.0%)		0 (0.0%)		
Menopausal status			14.89	<0.001		38.26	<0.001		0.489		0.626	
Premenopausal	236	104 (44.1%)			33 (14.0%)			1 (0.4%)		3 (1.3%)		
Postmenopausal	94	20 (21.3%)			43 (45.7%)			1 (1.1%)		2 (2.1%)		
Total	330	124 (37.6%)			76 (23.0%)			2 (0.6%)		5 (1.5%)	

P values for CIN2/3 and SCC were calculated using the Pearson chi-square test, whereas those for AIS and ADC were calculated using the Fisher–Freeman–Halton exact test. The two patients aged <25 years were described separately and excluded from the age-stratified inferential analysis because of the small sample size.

**Table 6:** Comparison of histopathological outcomes across alternative age cut-offs in women with ASC-H/HSIL cytology.

Age	Total	Glandular lesions						Glandular lesions			
		CIN2/3	*χ (^2^)*	*P*	SCC	*χ (^2^)*	*P*	AIS	*P*	ADC	*P*
30-year cut-off											
<30	27	17 (63.0%)	8.08	0.004	1 (3.7%)	6.20	0.013	0 (0.0%)	>0.999	0 (0.0%)	>0.999
≥30	303	107 (35.3%)			75 (24.8%)			2 (0.7%)		5 (1.7%)	
40-year cut-off											
<40	139	68 (48.9%)	13.18	<0.001	14 (10.1%)	22.75	<0.001	0 (0.0%)	0.511	2 (1.4%)	>0.999
≥40	191	56 (29.3%)			62 (32.5%)			2 (1.0%)		3 (1.6%)	
50-year cut-off											
<50	215	100 (46.5%)	21.00	<0.001	23 (10.7%)	52.94	<0.001	1 (0.5%)	>0.999	3 (1.4%)	>0.999
≥50	115	24 (20.9%)			53 (46.1%)			1 (0.9%)		2 (1.7%)	
60-year cut-off											
<60	288	120 (41.7%)	16.15	<0.001	48 (16.7%)	51.70	<0.001	2 (0.7%)	>0.999	5 (1.7%)	>0.999
≥60	42	4 (9.5%)			28 (66.7%)			0 (0.0%)		0 (0.0%)	

Analyses using different age cut-offs were exploratory sensitivity analyses. P values were two-sided and were not adjusted for multiple comparisons.

### 3.4 Multivariable logistic regression analysis of factors associated with CIN2+ in women with ASC-H/HSIL

A multivariable logistic regression model was constructed to evaluate factors associated with CIN2+ in women with ASC-H/HSIL cytology. As shown in **Table 7**, compared with hrHPV-negative women, HPV16 positivity was associated with markedly higher odds of CIN2+ (OR = 23.79, 95% CI: 10.67–53.08, P < 0.001). Similarly, HPV18/45 positivity (OR = 5.51, 95% CI: 1.53–19.86, P = 0.009) and positivity for the other 11 hrHPV types (OR = 4.26, 95% CI: 2.08–8.72, P < 0.001) were also independently associated with higher odds of CIN2 +. Age and menopausal status were not significantly associated with CIN2+ (both P > 0.05).

**Table 7:** Multivariable logistic regression analysis of factors associated with CIN2+ in women with ASC-H/HSIL cytology.

Variable	Category	β	SE	Wald χ²	P value	OR	95% CI for OR
Age (years)	—	-0.01	0.02	0.30	0.587	0.99	0.96–1.02
Menopausal status	No*	Reference	—	—	—	Reference	—
	Yes	0.22	0.45	0.25	0.621	1.25	0.52–3.03
hrHPV genotype	Negative*	Reference	—	—	—	Reference	—
	HPV16	3.17	0.41	59.94	<0.001	23.79	10.67–53.08
	HPV18/45	1.71	0.65	6.81	0.009	5.51	1.53–19.86
	Other 11 hrHPV types	1.45	0.37	15.64	<0.001	4.26	2.08–8.72

*control group.

Additional multivariable analyses (**Supplementary Tables S1**-**S4**) were performed to evaluate factors associated with specific pathological endpoints. For squamous lesions, HPV16 positivity (OR = 23.76, 95% CI: 10.62–53.15, P < 0.001) and positivity for the other 11 hrHPV types (OR = 4.60, 95% CI: 2.21–9.58, P < 0.001) were independently associated with higher odds of squamous HSIL +. In an exploratory analysis, HPV18/45 positivity was associated with AIS+; however, the estimate was imprecise, with a wide confidence interval (OR = 15.60, 95% CI: 1.42–171.32, P = 0.025), because of the small number of events. Younger age (OR = 0.93, 95% CI: 0.90–0.96, P < 0.001), HPV16 positivity (OR = 7.77, 95% CI: 3.30–18.32, P < 0.001), and positivity for the other 11 hrHPV types (OR = 6.76, 95% CI: 2.87–15.93, P < 0.001) were independently associated with CIN2/3. In contrast, increasing age (OR = 1.09, 95% CI: 1.05–1.14, P < 0.001) and HPV16 positivity (OR = 8.07, 95% CI: 2.57–25.34, P < 0.001) were independently associated with SCC.

## 4 Discussion

Cervical cancer screening serves as a critical component in the prevention and control of gynecologic malignancies. By identifying precancerous lesions and early-stage invasive cancers, it effectively interrupts disease progression, thereby achieving a significant reduction in incidence. Persistent infection with high-risk human papillomavirus (hrHPV) plays a pivotal role in cervical carcinogenesis. Cervical cytology combined with HPV testing has become the mainstream approach for cervical cancer screening. However, in some regions with lower Human Development Index (HDI), the lack of financial and technical capacity hinders the implementation of standardized screening programs. Meanwhile, HPV vaccination has not been widely implemented, and the challenges in cervical cancer screening and prevention remain formidable. As a country with a high HDI, China nevertheless faces regional disparities in development. Hebei Province, located in eastern China, is classified as a region with a medium HDI level, and therefore continues to face considerable pressure in cervical cancer screening and prevention.

In this study, we examined the prevalence of CIN2+ (CIN2, CIN3, AIS, SCC, or ADC) among women with ASC-H/HSIL cytology and evaluated its associations with hrHPV genotype, age, and menopausal status. Meanwhile, stratified analyses were performed according to AHPV genotype, age, and menopausal status to examine their associations with immediate histopathological outcomes. Overall, among women with ASC-H/HSIL cytology, 37.6% had CIN2/3, 23.0% had SCC, 0.6% had AIS, and 1.5% had ADC.

A study conducted in Anhui Province, China, in 2023 reported the CIN2+ detection rates among cases with abnormal cytology: ASC-US (18.6%), ASC-H (53.8%), LSIL (16.6%), HSIL (82.1%), SCC/ADC (100.0%), and AGC (63.8%) (11). Similar studies have shown that the CIN2+ detection rate among women with HSIL cytology was 89.6% in the Korean population (22), and another domestic study confirmed a CIN2+ detection rate of 82.6% within 6 months after HSIL diagnosis (23). For the population with ASC-H cytology, a German study demonstrated that approximately 50.7% of patients exhibited significant pathological results (CIN2+) (24); moreover, a 2023 study by Juan Li et al. (25) reported an HSIL+ detection rate of 64.9% in patients with ASC-H cytology. In the present study, the squamous HSIL+ detection rates among women with ASC-H and HSIL cytology were 39.1% and 83.2%, respectively. Factors contributing to these discrepancies may include differences in testing methods, variations in regional HDI levels, disparities in educational attainment among the screened populations, and the increased uptake of cervical cancer screening in recent years.

A meta-analysis of cervical infection-to-cancer progression showed that the hrHPV infection rate in HSIL was highest in Oceania (98%) and lowest in Western/Central Asia (72%), with an overall infection rate of 85% (26). Furthermore, the performance of hrHPV testing is highly dependent on laboratory techniques. Previous studies have confirmed that AHPV testing demonstrates higher sensitivity than other first-line screening methods in high-risk populations (6). Marked differences exist in the carcinogenic characteristics of different HPV subtypes: HPV16 is associated with progressive carcinogenic pathways, whereas HPV18 often complicates the identification of precancerous lesions. Clinical data from China indicate that 69.1% of cervical cancer cases are attributable to HPV16/18 infection (27, 28). This study, conducted in Hebei Province, employed AHPV testing and revealed an hrHPV positivity rate of 94.4% in the HSIL population. This prevalence differs from that reported in previous international studies and may reflect regional differences in HPV epidemiology. Analysis of the HSIL population in this study showed a CIN2+ detection rate of 84.2% among hrHPV-positive cases, which is largely consistent with the findings of the US-based study by Aofei Li et al. (7). Concurrently, the hrHPV negativity rate in this population was 5.6% (9/161), which aligns closely with the 5%–8% range reported in domestic and international literature (29, 30). Immediate histopathological comparison revealed that the CIN2/3 prevalence in the hrHPV-positive group was significantly higher than that in the negative group (*P* < 0.05). Notably, among the nine women with HSIL cytology but negative hrHPV results, six were histopathologically confirmed as having CIN2+ (66.7%), a proportion comparable to that reported in a previous study (31). Among the 59 hrHPV-negative women with ASC-H/HSIL cytology, histopathology identified squamous HSIL+ in 12 women (20.3%) and AIS+ in one woman (1.7%), yielding an overall prevalence of clinically significant lesions of 22.0%. These data confirm that a substantial proportion of occult high-grade cervical lesions and early carcinomas exist among individuals with high-grade cytological abnormalities but negative hrHPV results, underscoring important clinical vigilance and unequivocally demonstrating the non-negligible risk of missed diagnosis inherent in a single hrHPV screening paradigm. Integrating the findings of this study with existing literature, the core reasons for detecting high-grade cervical lesions despite negative hrHPV results can be summarized as follows: Several possible explanations may account for the detection of high-grade cervical lesions in women with negative hrHPV results, including inadequate sampling, E6/E7 mRNA expression below the assay detection threshold, infection with HPV genotypes outside the assay target panel, or persistence of established lesions after viral clearance. However, specimen adequacy, viral mRNA levels, non-target HPV genotypes, and previous HPV infection or clearance were not directly evaluated in this study. Therefore, these explanations remain hypothetical, and the underlying mechanisms cannot be determined from the present data. This fully indicates that clinicians must not exclude high-grade cervical lesions solely based on negative HPV results. For high-risk populations with ASC-H/HSIL cytology, regardless of HPV test outcomes, colposcopy and targeted biopsy must be performed according to standardized protocols to circumvent missed or erroneous diagnoses and delayed early treatment due to the limitations of single-modality screening, thereby highlighting the core clinical significance of combined screening in the early detection and diagnosis of cervical lesions. Additionally, subgroup analysis revealed that the prevalence of CIN2/3 among individuals positive for HPV-16 and the other 11 high-risk HPV subtypes was significantly higher than that in the negative population (*P* < 0.001).

Cervical cancer is predominantly SCC, with ADC accounting for 10%–20% of all cervical cancers globally (32). Compared with SCC of the same stage and tumor size, ADC carries a poorer prognosis due to its higher metastasis rate (33). In recent years, the incidence of SCC has shown a declining trend in high-HDI countries such as the United States and Europe, whereas the incidence of ADC has relatively increased (34). This rise is attributed to an increased incidence among younger women (35). On conventional Pap smears, the cytological features of AIS are subtle and frequently overlap with those of CIN; however, liquid-based cytology enhances the sensitivity for detecting glandular lesions (36). HSIL often serves as a “sentinel” cytological manifestation of AIS (37); nonetheless, the prevalence of AIS/ADC in cervical cytology showing HSIL is approximately 2%–3% (38), and can be as high as over 13.8% in HPV-18/45-positive populations (14). Multiple studies have confirmed marked heterogeneity in the distribution of different hrHPV genotypes across various cervical malignant lesions (39): HPV16 predominates in squamous epithelial lesions, whereas HPV18 is more frequently associated with glandular epithelial lesions (40). In this study, comparison of AIS detection rates across different hrHPV genotypes revealed that the AIS detection rate in the HPV-18/45-positive group was 15.4%, while no AIS lesions were detected in the other genotype groups, and the difference was statistically significant (P = 0.001). In this study, both AIS cases occurred among the 13 HPV18/45-positive women. Although the exploratory multivariable analysis suggested an association between HPV18/45 positivity and AIS+, the estimate was highly imprecise, with a wide confidence interval (OR = 15.60, 95% CI: 1.42–171.32), because only two AIS cases were identified. Therefore, this finding should be considered hypothesis-generating and does not support genotype-specific diagnostic or interventional recommendations. Confirmation in larger cohorts is required. Existing evidence suggests that combined cytology and HPV screening holds greater utility in detecting cervical cancer, especially cervical adenocarcinoma and its precursor lesions (37).

This study included 330 women with ASC-H/HSIL cytology, with an overall hrHPV positivity rate of 82.1%. The hrHPV positivity rate was significantly higher among postmenopausal women than among premenopausal women (91.5% vs. 78.4%). This finding differs from that of a previous study, which found no overall difference in hrHPV positivity between premenopausal and postmenopausal women, although higher positivity was observed among women aged 65 years or older (41). The higher hrHPV positivity observed among postmenopausal women in the present study may be related to age-associated hormonal changes, immune senescence, or reactivation of latent HPV infection. However, hormonal levels, immune function, and latent viral reactivation were not evaluated; therefore, these mechanisms remain possible explanations rather than conclusions derived from the present data. These factors may impair the body’s ability to spontaneously clear HPV, facilitate the reactivation of latent infections, and consequently increase the probability of clinically apparent infections. Previous research by Juan Li et al. demonstrated that the incidence of CIN2+ in young women aged <40 years was significantly higher than that in women aged ≥40 years (25), and another independent study suggested variations in HSIL+ risk across different age groups (42). The results of this study indicated a significant downward trend in the CIN2/3 detection rate with advancing age, whereas the SCC detection rate increased correspondingly. This is consistent with the findings of Yang J et al. in China (43). This age-related pattern may reflect differences in previous screening exposure, treatment history, lesion persistence or progression, and cervical transformation-zone visibility. However, individual screening histories, previous treatment, and transformation-zone characteristics were not evaluated in this study; therefore, these explanations remain speculative and cannot be confirmed by the present data. The present findings are generally consistent with previous studies and further support the current ASCCP recommendations regarding risk-based management of women with ASC-H/HSIL cytology.

This study has several limitations. First, its retrospective, single-center design may limit the generalizability of the findings. Inclusion also required the availability of both Aptima HPV testing and histopathological confirmation, which may have preferentially selected women who completed further diagnostic evaluation or had more concerning clinical findings. This selection and verification bias may have overestimated lesion prevalence and limits the applicability of the findings to all women with ASC-H/HSIL cytology. Second, excisional or surgical specimens were not available for all women. Although the highest histopathological grade across all available specimens was used as the final diagnosis, reliance on colposcopy-directed biopsy alone in some women may have resulted in underestimation of CIN2+ lesions. Centralized rereview of all histopathological slides was not performed, and some interobserver variability cannot be excluded. Finally, the HPV18/45-positive subgroup was small (n = 13), and only two AIS cases were identified, resulting in a highly imprecise estimate with a wide confidence interval. The observed association between HPV18/45 positivity and glandular lesions should therefore be regarded as exploratory and requires confirmation in larger cohorts.

## 5 Conclusion

This study showed that immediate histopathological outcomes among women with ASC-H/HSIL cytology differed according to hrHPV genotype. HPV16 positivity was strongly associated with cervical precancerous and malignant lesions. Age and menopausal status showed differences in the unadjusted distributions of squamous lesions but were not independently associated with CIN2 +. The possible association between HPV18/45 positivity and glandular lesions requires confirmation in larger cohorts.

## Data Availability

The original contributions presented in the study are included in the article/**Supplementary Material**. Further inquiries can be directed to the corresponding author.
