# Supplementary material for: Immediate histopathological outcomes according to Aptima HPV E6/E7 mRNA genotype, age, and menopausal status in women with ASC-H/HSIL cytology
Source: Front Oncol. 2026 Oct 26;16:1952454. doi: 10.3389/fonc.2026.1952454 (PMC13745243; doi:10.3389/fonc.2026.1952454)
Supplement: Supplementary file 1 [file Table1.docx]

**Supplementary Table S1.** Multivariable Logistic Regression Analysis of Factors Associated with HSIL+ in Women with ASC-H/HSIL.

| **Variable** | **Category** | **β** | **SE** | **Wald χ²** | **P value** | **OR** | **95% CI for OR** | |
| --- | --- | --- | --- | --- | --- | --- | --- | --- |
|  |  |  |  |  |  |  | **Lower** | **Upper** |
| Age (years) | — | -0.01 | 0.02 | 0.28 | 0.594 | 0.99 | 0.96 | 1.02 |
| Menopausal status | No* | Reference | — | — | — | Reference | — | — |
|  | Yes | 0.13 | 0.45 | 0.09 | 0.763 | 1.14 | 0.48 | 2.74 |
| hrHPV genotype | Negative* | Reference | — | — | — | Reference | — | — |
|  | HPV16 | 3.17 | 0.41 | 59.48 | <0.001 | 23.76 | 10.62 | 53.15 |
|  | HPV18/45 | 0.88 | 0.66 | 1.79 | 0.181 | 2.42 | 0.66 | 8.79 |
|  | Other 11 hrHPV types | 1.53 | 0.37 | 16.63 | <0.001 | 4.60 | 2.21 | 9.58 |

Note: * Reference (control) group.

**Supplementary Table S2.** Multivariable Logistic Regression Analysis of Factors Associated with AIS+ in Women with ASC-H/HSIL.

| **Variable** | **Category** | **β** | **SE** | **Wald χ²** | **P value** | **OR** | **95% CI for OR** | |
| --- | --- | --- | --- | --- | --- | --- | --- | --- |
|  |  |  |  |  |  |  | **Lower** | **Upper** |
| Age (years) | — | 0.00 | 0.05 | 0.00 | 0.966 | 1.00 | 0.90 | 1.11 |
| Menopausal status | No* | Reference | — | — | — | Reference | — | — |
|  | Yes | 0.65 | 1.32 | 0.24 | 0.622 | 1.92 | 0.14 | 25.53 |
| hrHPV genotype | Negative* | Reference | — | — | — | Reference | — | — |
|  | HPV16 | -0.35 | 1.26 | 0.08 | 0.779 | 0.70 | 0.06 | 8.32 |
|  | HPV18/45 | 2.75 | 1.22 | 5.05 | 0.025 | 15.60 | 1.42 | 171.32 |
|  | Other 11 hrHPV types | -0.82 | 1.43 | 0.33 | 0.569 | 0.44 | 0.03 | 7.32 |

Note: * Reference (control) group.

**Supplementary Table S3.** Multivariable Logistic Regression Analysis of Factors Associated with CIN2/3 in Women with ASC-H/HSIL.

| **Variable** | **Category** | **β** | **SE** | **Wald χ²** | **P value** | **OR** | **95% CI for OR** | |
| --- | --- | --- | --- | --- | --- | --- | --- | --- |
|  |  |  |  |  |  |  | **Lower** | **Upper** |
| Age (years) | — | -0.07 | 0.02 | 17.80 | <0.001 | 0.93 | 0.90 | 0.96 |
| Menopausal status | No* | Reference | — | — | — | Reference | — | — |
|  | Yes | 0.03 | 0.43 | 0.01 | 0.942 | 1.03 | 0.44 | 2.42 |
| hrHPV genotype | Negative* | Reference | — | — | — | Reference | — | — |
|  | HPV16 | 2.05 | 0.44 | 21.97 | <0.001 | 7.77 | 3.30 | 18.32 |
|  | HPV18/45 | 0.18 | 0.88 | 0.04 | 0.841 | 1.19 | 0.21 | 6.74 |
|  | Other 11 hrHPV types | 1.91 | 0.44 | 19.07 | <0.001 | 6.76 | 2.87 | 15.93 |

Note: * Reference (control) group.

**Supplementary Table S4.** Multivariable Logistic Regression Analysis of Factors Associated with SCC in Women with ASC-H/HSIL.

| Variable | Category | β | SE | Wald χ² | P value | OR | 95% CI for OR | |
| --- | --- | --- | --- | --- | --- | --- | --- | --- |
|  |  |  |  |  |  |  | Lower | Upper |
| Age (years) | — | 0.09 | 0.02 | 20.08 | <0.001 | 1.09 | 1.05 | 1.14 |
| Menopausal status | No* | Reference | — | — | — | Reference | — | — |
|  | Yes | -0.14 | 0.47 | 0.08 | 0.772 | 0.87 | 0.34 | 2.21 |
| hrHPV genotype | Negative* | Reference | — | — | — | Reference | — | — |
|  | HPV16 | 2.09 | 0.58 | 12.79 | <0.001 | 8.07 | 2.57 | 25.34 |
|  | HPV18/45 | 1.47 | 0.89 | 2.71 | 0.100 | 4.35 | 0.76 | 25.07 |
|  | Other 11 hrHPV types | -0.01 | 0.65 | 0.00 | 0.990 | 0.99 | 0.28 | 3.53 |

Note: * Reference (control) group.
